# Coherences in the Dynamics of Physical Systems from a Multifractal Perspective of Motion

**DOI:** 10.3390/e25081143

**Published:** 2023-07-30

**Authors:** Decebal Vasincu, Andreea Bianca Bruma, Oana Rusu, Cristina Marcela Rusu, Vlad Ghizdovat, Maricel Agop

**Affiliations:** 1 Faculty of Dental Medicine, “Grigore T. Popa” University of Medicine and Pharmacy, 700115 Iasi, Romania; decebal.vasincu@umfiasi.ro; 2 Faculty of Physics, ”Alexandru Ioan Cuza” University of Iasi, 700506 Iasi, Romania; bianca_andreea_bruma@yahoo.com; 3 Faculty of Material Science and Engineering, “Gheorghe Asachi” University of Iasi, 700050 Iasi, Romania; oana.rusu@academic.tuiasi.ro; 4 Department of Physics, “Gheorghe Asachi” Technical University of Iasi, 700050 Iasi, Romania; cristina.rusu@tuiasi.ro; 5 Department of Biophysics and Medical Physics, “Grigore T. Popa” University of Medicine and Pharmacy, 700115 Iasi, Romania; 6 Romanian Scientists Academy, 54 Splaiul Independentei, 050094 Bucharest, Romania

**Keywords:** fractality, scale relativity theory, Ricatti gauge, special ansatz, multifractal Schrödinger equation

## Abstract

Using an analogy between the multi-fractal Schrödinger equation and the dumped oscillator equation through a special ansatz, Stoler-type coherences in the dynamics of physical systems are highlighted. Such a result implies a Ricatti-type gauge, a process that can be considered a calibration of the difference between the kinetic and potential energy of a Lagrangian, specified as a perfect square in generic coordinates.

## 1. Introduction

Unlike classic theories (fluid mechanics, General Relativity Theory, etc.), in which the dynamics of the physical systems are described through continuous and differentiable curves, the Scale Relativity Theory [1,2,3] operates in the description of the dynamics of physical systems with continuous and non-differentiable curves (fractal/multifractal curves). Because any fractal/multifractal curve exhibits the property of self-similarity in any one of its points (every part reflects the whole and vice versa), the holographic-type implementations are thus fundamental in the description of the dynamics of physical systems [4,5,6]. In such a context, instead of operating with a single variable (a strictly non-differentiable mathematical function), it is possible to operate only with approximations of the mathematical function, with said approximations resulting from averaging it at different-scale resolutions. Thus, any physical variable used to describe the dynamics of complex systems will operate as the limit of a family of mathematical functions, with the function being non-differentiable for zero-scale resolution and differentiable for non-zero scale resolution.

In such an approach, many dynamical problems have been analyzed, both at a small scale (Kepler-type problem in the fractal hydrodynamic model, harmonic oscillator-type problem in the fractal hydrodynamic model, elastic- and plastic-type behaviors in the fractal theory of motion, etc. [2,3,7,8,9,10,11]) and at a large scale (solar system dynamics, Hubble effect, etc. [4]).

In the present paper, using an analogy between the multifractal Schrödinger equation and the dumped oscillator equation through a special ansatz, Stoler-type coherences in the dynamics of physical systems are highlighted.

## 2. Correspondences between Multifractal Schrödinger Equation and Dumped Oscillator through a Special Ansatz

The multifractal Schrödinger equation is considered for a constant scalar potential U [2,3]:

(1) $$\alpha^{2}\partial_{l}\partial^{l}\Psi +i\alpha \partial_{t}\Psi +\frac{U}{2}\Psi =0$$

where

(2) $$\alpha =\lambda {\left(dt\right)}^{\left[\frac{2}{f\left(\alpha \right)}\right]-1}$$

and

(3) $$\partial_{l}=\frac{\partial }{\partial x^{l}},\;\;\partial_{l}\partial^{l}=\frac{\partial }{\partial x^{l}}\frac{\partial }{\partial x^{l}},\;\;\partial_{t}=\frac{\partial }{\partial_{t}},\;\;\;\;i,l=1,2,3$$

The quantities from Equations (1)–(3) have the following meaning: $x^{l}$ is a non-differentiable spatial coordinate, *t* is a differentiable temporal coordinate with the role of an affine parameter of the non-differentiable motion curves (fractal/multifractal curves), Ψ is the state function, *U* is the external scalar potential, *dt* is the resolution scale, λ is a constant coefficient associated with the differentiable–non-differentiable scale transition, and $f\left(\alpha \right)$ is the singularity spectrum of order α, where $\alpha =\alpha \left(D_{F}\right)$ and $D_{F}$ is the fractal dimension of the motion curves. For details see [2,3].

By admitting the functionality of the ansatz [6,12]:

(4) $$\xi =a_{1}x+a_{2}y+a_{3}z-ia_{4}t$$

Equation (1) with substitutions:

(5) $$M=\alpha^{2}\left({a_{1}}^{2}+{a_{2}}^{2}+{a_{3}}^{2}\right),\;\;2R=\alpha a_{4},\;\;K=\frac{U}{2},\;\;q\equiv \Psi$$

is formally reduced to a damped oscillator, i.e.,

(6) $$M\ddot{q}+2R\dot{q}+Kq\equiv 0$$

We must mention that the meaning of ansatz (4) will be given at a later time.

In what follows, the physical significances will first be used for the classic case (i.e., for the damping oscillator) and then for the SRT case.

Regarding the classic case, q is the relevant coordinate of the motion. We prefer this equation to other formalisms from several points of view, but mainly due to the fact that the physical parameters that characterize the harmonic oscillator—the coefficients of the equation—have always had in history, often even explicitly, an explanation through interaction. R is the motion damping coefficient, and it is a characteristic of the force—the proportionality with the instantaneous speed—that opposes instantaneous motion. *K*-elasticity or elastic stiffness, although usually considered as an intrinsic property of the oscillator, is actually a property of the structure for which the harmonic oscillator is a physical component, i.e., of the local universe, and as such is also an interaction property. The same can be said about mass *M*, only that the fact is not so obvious: the history of physics still has considerations regarding different types of masses, determined by different types of interactions that a particle can support (electromagnetic mass, gravitational mass, inertial mass, etc.). In Equation (6) it is assumed that we have to work with inertial mass. As such, we retain here the idea that mass is an expression of global circumstances of the existence of matter, as elastic stiffness is an expression of local circumstances.

The circumstances, always left unspecified in such a problem, are therefore summarized in the explanation of these physical parameters by interactions. In the usual problems we are only interested in their fixed values, and these values intuitively explain the movement described by Equation (6). However, there are situations that require an explanation of these parameters by interaction and, when physics comes back for such an explanation, it is without exception either completely unsatisfactory or, at best, partially. We want to put things in order, at least as far as the place of this physical explanation is concerned. We are sure that only after this, things will naturally settle in their natural places. The dynamics analysis that follows takes into consideration the mathematical methodology from [13].

Equation (6) can be written as a system:

(7) $$\dot{p}=-2\frac{R}{M}p-\frac{K}{M}q;\dot{q}=p$$

The second of these equations is obviously only a definition of momentum. System (7) is not yet a Hamiltonian system, as would be expected when discussing about momentum and coordinate, because its matrix is not an involution (it has no zero trace). This fact is much more obvious if we put the system in matrix form:

(8) $$\left(\begin{array}{c} \dot{p}\\ \dot{q} \end{array}\right)=\left(\begin{array}{cc} -2\frac{R}{M} & -\frac{K}{M}\\ 1 & 0 \end{array}\right)\left(\begin{array}{l} p\\ q \end{array}\right)$$

As long as the physical parameters contained in the 2×2 matrix of this system are constant, we can put the system in an equivalent form that highlights the position of the energy, and so of the Hamiltonian system (of course, in the particular instances where it can be identified with energy). Indeed, from (8) we can immediately obtain the equation

(9) $$\frac{1}{2}M(p\dot{q}-q\dot{p})=\frac{1}{2}\left(MP^{2}+2Rpq+Kq^{2}\right)$$

which proves that the energy—the quadratic form on the right—is the rate of variation of the physical action represented by the elementary area in the phase plane (q,p). This was, incidentally, always the case in physics, so Equation (9) does not represent anything new. However, we want to emphasize here the fact that energy is not required to be conserved in order to be taken as the rate of a physical action. All that is required is that the action be adequately defined as an area in the phase plane. Equation (9) is actually a Riccati-type equation for a certain frequency, because it can be put into the shape

(10) $$M\dot{\omega }+M\omega^{2}+2R\omega +K=0;\omega =\frac{p}{q}$$

It is not necessary to ask now whether this frequency has a physical meaning or not. We note only that the solution of the above equation is given by the ratio of the solutions of the Hamiltonian system corresponding to (8), i.e.,

(11) $$\left(\begin{array}{c} \dot{p}\\ \dot{q} \end{array}\right)=\left(\begin{array}{cc} -\frac{R}{M} & -\frac{K}{M}\\ 1 & \frac{R}{M} \end{array}\right)\left(\begin{array}{l} p\\ q \end{array}\right)$$

This is, moreover, a general characteristic of the relationship between Riccati’s equation and Hamiltonian dynamics [14]. We can return to Equation (9) above by simply constructing from (11) the 1-differential form that characterizes the elementary area in the phase plane. As far as Equation (10), it can be easily integrated to show us that energy is no longer conserved, but we have the much more complicated conservation law discovered by H. H. Denman [15].

(12) $$\begin{array}{rr} & \frac{1}{2}\left(Mp^{2}+2Rpq+Kq^{2}\right)\cdot \exp\left\{\frac{2R}{\sqrt{MK-R^{2}}}\right.\\ & \left.\times \tan^{-1}\left(\frac{Mp+Rq}{q\sqrt{MK-R^{2}}}\right)\right\}=\mathrm{const}. \end{array}$$

It can be seen from here that energy is conserved in the classical sense only if the coefficient of damping is zero or the movement in the phase plane is made on a straight line passing through the origin, with the slope determined by the ratio between the damping coefficient and the mass. Cancellation of the damping coefficient is usually associated with the absence of dissipative forces. This makes physical sense, but our chief concern here is the identity of these dissipative forces. As such, we will focus on the significance of the Riccati equation, Equation (10), and of the associated Hamiltonian system (11).

What would be the possibility of “agreement” required for the measurement? Note that the classical equation of motion (6) is the expression of a variational principle related to the Lagrangian

(13) L(q,q˙,t)=12Mq˙2−Kq2exp2RTM

which represents a harmonic oscillator with explicitly time-varying parameters. Integral on one finite interval $\left[t_{0},t_{1}\right]$ of this function is the physical action of the oscillator on that interval of time, i.e., the difference between the average kinetic energy and the average potential energy. For obtaining Equation (6) it is necessary to take the variation in this action under the mandatory condition that the variation in the coordinate at the ends of the time interval should be zero:

(14) $${\left.\delta q\right|}_{t_{0}}={\left.\delta q\right|}_{t_{1}}=0$$

However, to obtain a closed trajectory, we need to ask even more, as the coordinate values at the ends of the time interval should be the same:

(15) $$q\left(t_{0}\right)=q\left(t_{1}\right)$$

If this trajectory must be closed in the phase plane, it is obvious that the values of the speed at the ends of the time interval must be the same. These circumstances allow us to define the measurement process with the help of the harmonic oscillator in a slightly more precise way. Indeed, let us direct our thought along the following line: from the point of view of the variational principle and the resulting equation of motion, the Lagrangian (13) is defined up to an additive function to be time derivative of another function. The process is extensively used in various branches of theoretical physics to define the so-called gauge transformations. Let us then proceed as usual and define a gauge in whose Lagrangian is a perfect square. This fact is well known and widely exploited in control theory [14], so that we are not in unknown territories. The procedure is to add to the Lagrangian (13) the term

(16) $$\frac{1}{2}\frac{d}{dt}\left[w\exp\left(\frac{2R}{M}t\right)Q^{2}\right]$$

where w is a continuous function of time, requiring that the final Lagrangian be a perfect square. The variation of the function under the derivative operator is zero due to the conditions in Equation (14), so that the equation of motion does not change. The new Lagrangian, expressed in generic coordinates, turns out to be

(17) L(Q,Q˙,t)=12Mexp2RtMQ˙+wMQ2

provided that w satisfies the following Riccati-type differential equation:

(18) $$\dot{w}=\frac{1}{M}w^{2}-2\frac{R}{M}w+K$$

The Lagrangian (17) will be taken here to represent the energy of the measurement results. As before, there is a relationship between the Riccati equation, Equation (18), and the Hamiltonian dynamics. We find that the analog of Equation (11) is

(19) $$\left(\begin{array}{c} \dot{\eta }\\ \dot{\xi } \end{array}\right)=\left(\begin{array}{cc} -\frac{R}{M} & \frac{K}{M}\\ -1 & \frac{R}{M} \end{array}\right)\left(\begin{array}{c} \eta \\ \xi \end{array}\right);w\equiv \frac{\eta }{\xi }$$

which obviously represents a Hamiltonian system. Therefore, we can, strictly speaking, conveniently identify the factors of w with the coordinates in the phase plane. It can be said that “the Lagrangian of measurement” (17) represents a set of oscillators along a certain Hamiltonian evolution in the phase plane, an evolution given by Equation (19). The problem now is the physical meaning of this assembly. However, before giving an answer to this problem, a little digression is in order.

One can ask the natural question: what is so special about the quadratic Lagrangian that represents the measurement results? We have no other answer than a philosophical one, deduced from our theoretical experience. First let us recall that free particle mechanics can be described by considering the kinetic energy as a Lagrangian—a quadratic Lagrangian—which leads directly to the equations of motion of the free particle [16]. Therefore, we say, why not reverse the process, and say that a quadratic Lagrangian actually represents an elementary physical structure, like the harmonic oscillator, as free as its circumstances permit. In order to be able to say, “this oscillator made this measurement”, we must be able first to discern that oscillator as a stand-alone structure within the physics structure of which it is a part. When we refer to the classical free particle, we identify it by the fact that it only has kinetic energy—and so is a quadratic Lagrangian—so we will indicate a harmonic oscillator by a quadratic Lagrangian leading to its equations of motion. Therefore, such a Lagrangian will be taken as representing the measurement with the harmonic oscillator. This purely philosophical conclusion will finally be demonstrated rationally with the help of Stoka’s theorem.

Equation (18) shows us that w is a dissipation coefficient, more precisely a variation rate of the mass (in the case that mass is not constant), and this mass variation refers to the oscillator harmonic that performs the measurement. For obvious physical reasons it is therefore important to find the most general solution of that equation. José Carineña and Arturo Ramos offer us a pass in a short but modern and pertinent review of the integrability of Riccati’s equation [16,17]. For our current needs it is enough to note that the complex numbers

(20) $$w_{0}\equiv R+iM\Omega ,w_{0}^{*}\equiv R-iM\Omega ;\Omega^{2}=\frac{K}{M}-{\left(\frac{R}{M}\right)}^{2}$$

are the roots of the quadratic polynomial on the right side of Equation (18). Therefore, first we perform the homographic transformation:

(21) $$z=\frac{w-w_{0}}{w-w_{0}^{*}}$$

and now it can easily be seen by direct calculation that z is a solution of the linear and homogeneous equation of the first order

(22) $$\dot{z}=2i\Omega z∴z(t)=z(0)e^{2i\Omega t}.$$

Therefore, if we conveniently express the initial condition z(0), we can give the general solution of the Equation (18) by simply inverting the transformation (21), with the result

(23) $$w=\frac{w_{0}+re^{2i\Omega \left(t-t_{r}\right)}w_{0}^{*}}{1+re^{2i\Omega \left(t-t_{r}\right)}}$$

where r and $t_{r}$ are two real constants that characterize the solution. Using Equation (20) we can put this solution in real terms, i.e.,

(24) $$\begin{array}{rr} z= & R+M\Omega \left(\frac{2r\sin\left[2\Omega \left(t-t_{r}\right)\right]}{1+r^{2}+2r\cos\left[2\Omega \left(t-t_{r}\right)\right]}\right.\\ & \left.+i\frac{1-r^{2}}{1+r^{2}+2r\cos\left[2\Omega \left(t-t_{r}\right)\right]}\right) \end{array}$$

which highlights a frequency modulation through what we would call a Stoler transformation (coherences of Stoler type) [18,19] and leads us to a complex form of this parameter. More than that, if we make the notation

(25) r≡coth⁡τ

Equation (24) becomes

(26) z=R+MΩh

where h is given by

(27) $$h=-i\frac{\cosh\tau -e^{-2i\Omega \left(t-t_{m}\right)}\sinh\tau }{\cosh\tau +e^{-2i\Omega \left(t-t_{m}\right)}\sinh\tau }$$

Now, through Equation (27), correspondences between the ansatz (4) and the concept of skyrmions can be found (see Appendix A). We note that *h* from (27) appears as a solution of the equations

$$\left(h-h^{*}\right)\nabla \left(\nabla h\right)=2{\left(\nabla h\right)}^{2}\;\left(h-h^{*}\right)\nabla \left(\nabla h^{*}\right)=2{\left(\nabla h^{*}\right)}^{2}$$

equations which result through harmonic mappings of the Poincaré metric

(28) $$ds^{2}=\frac{dhdh^{*}}{{\left(h-h^{*}\right)}^{2}}=\frac{(du{)}^{2}+(dv{)}^{2}}{v^{2}}$$

For details, see ref. [6] from Appendix A.

Moreover, we must mention that a Hamiltonian theory can be built for h=u+iv. For details, see Appendix B.

**Figure 1:**
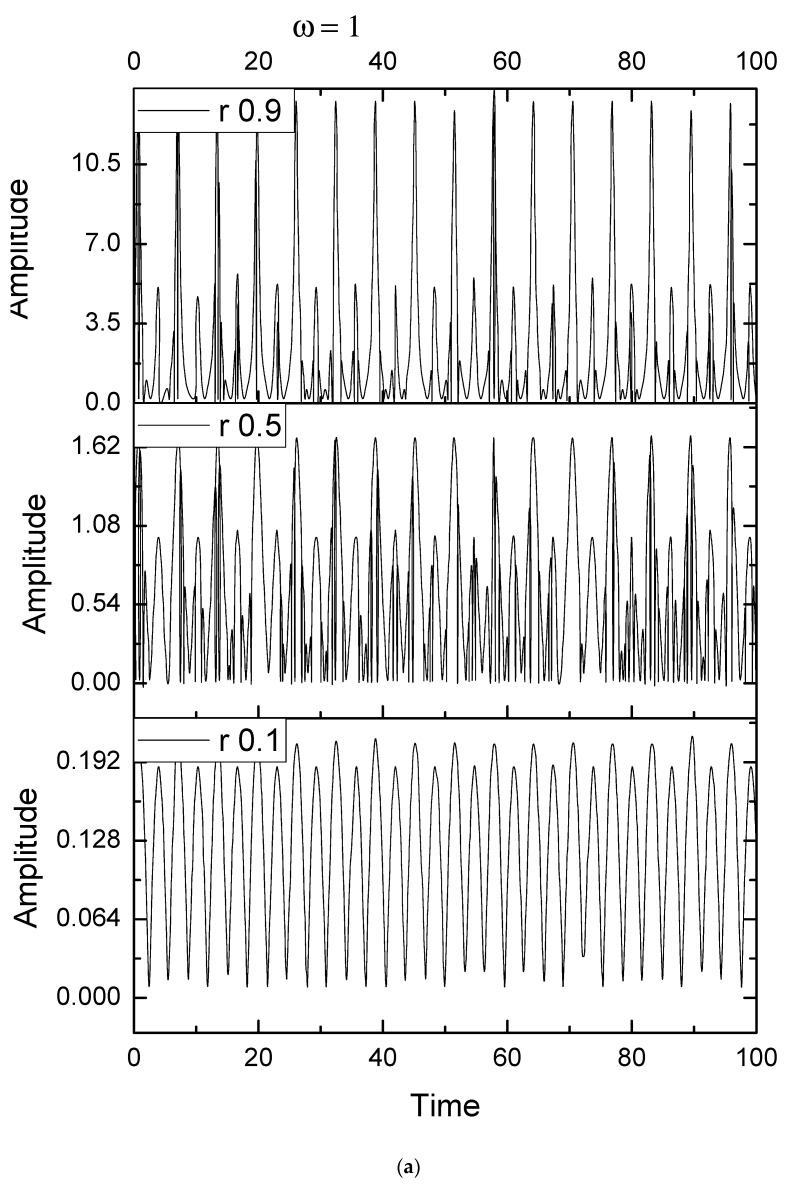

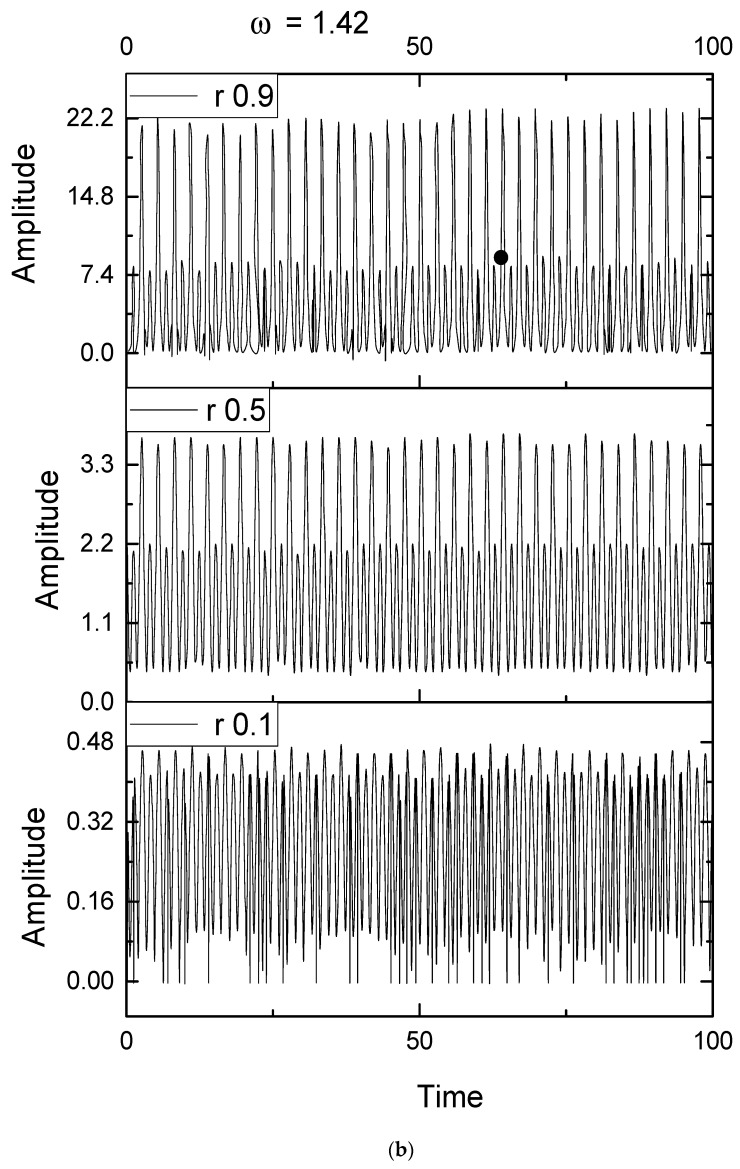

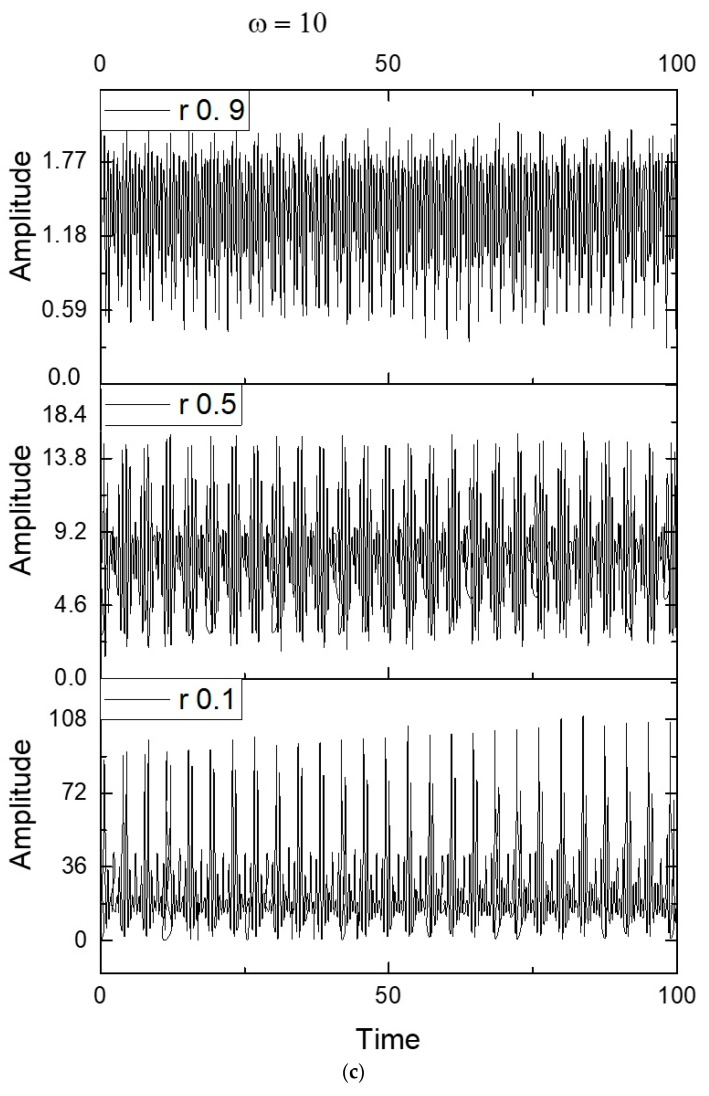

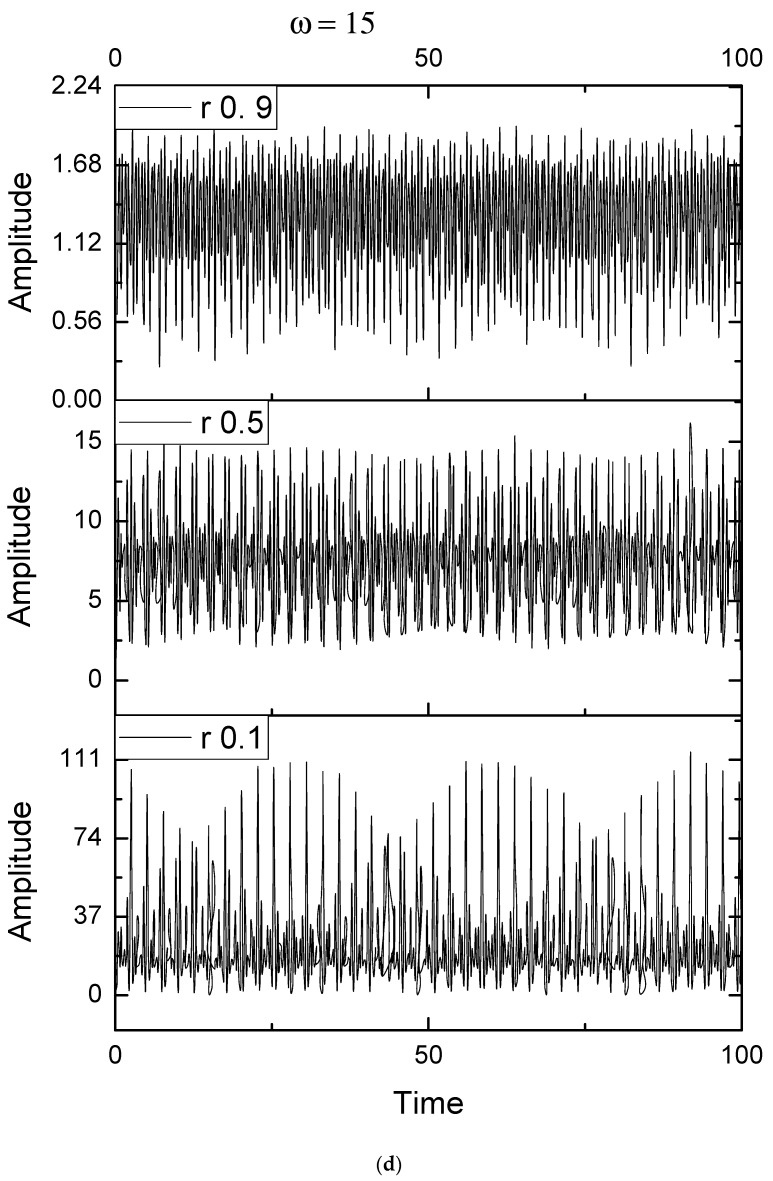
Stoler-type coherences by means of |*h*| for different values of the pulsation-type characteristic *ω* (1, 1.42, 10, and 15) and for three different *r* values (0.1, 0.5, and 0.9) in the form of period-doubling (**a**), damped oscillations (**b**), quasi-periodicity (**c**), and intermittences (**d**).

Taking into account that *h* is the solution for the above-presented equations, in Figure 1a–d we show this solution, which can be associated to various Stoler-type coherences, in the form of period-doubling (Figure 1a), damped oscillations (Figure 1b), quasi-periodicity (Figure 1c), and intermittences (Figure 1d). These figures were obtained by means of |*h*| for different values of the pulsation-type characteristic *ω* (1, 1.42, 10, and 15) and for three different *r* values (0.1, 0.5, and 0.9). The period-doubling and quasi-periodicity behaviors can be verified by means of the Fast Fourier Transform.

Let us note that the measurement process appears here as a frequency modulation process. More precisely, this process is a calibration of the difference between kinetic and potential energy—the Lagrangian classic definition—which brings this quantity to a perfect square. The physical meaning of the perfect square Lagrangian is that it describes a fundamental physical unit in the interior of a complicated system, as kinetic energy describes the free particle in space. As expected, the quadratic Lagrangian actually corresponds to an ensemble of fundamental physical units: a set of oscillators of the same frequency.

## 3. Conclusions

The main conclusions of the present paper are the following:

i. An analogy between the multifractal Schrödinger equation and the dumped oscillator equation through a special ansatz is established;
ii. Using a Ricatti-type gauge, Stoler-type coherences in the dynamics of physical systems are highlighted;
iii. This Ricatti-type gauge was assimilated to a calibration process of the difference between the kinetic and the potential energy of a Lagrangian, specified as a perfect square in generic coordinates.

## Appendix A

The skyrmion is a topological soliton that can represent, in appropriate conditions, the nucleons, as forms of the nuclear matter. This concept was first developed by Tony Skyrme in [20,21]. Later, in the same framework, Slobodeanu [22] focused on maps from the usual space to the hyperbolic space.

As is known, such a harmonic map is obtained by finding the functions which realize the extremum of an *energy* functional of the form

(A1) $$E_{2}\left(\Phi \right)=∭\left\langle d\Phi |d\Phi \right\rangle d^{3}\vec{x}$$

where $d^{3}\vec{x}$ is the volume element of space. Here, the function realizing the correspondence is, generally speaking, of the nature of a matrix, and this is why we denoted it by a bold Φ. Its differential actually represents a space gradient of the correspondence in the current metric of the ambient physical space—the Euclidean metric space. On the other hand, the internal product from the functional (A1) is the one induced by the metric of the hyperbolic plane or space. In the case of Kepler motion, for instance, we may write

(A2) $$\Phi \equiv \left(\begin{array}{cc} h+h^{*} & -2hh^{*}\\ 2 & -h-h^{*} \end{array}\right);\;\left\langle d\Phi |d\Phi \right\rangle \equiv \frac{-4\nabla h\cdot \nabla h^{*}}{{\left(h-h^{*}\right)}^{2}}$$

where h is the complex number defined in Equation (27), comprising the eccentricity and the orientation of the orbit, and $h^{*}$ is its complex conjugate. In other words, the Kepler motion is represented by the matrix of its quadratic form, written in terms of its asymptotic directions. The metric in (A2) is then the absolute metric from [2,3]. At the risk of repeating things well known, it is perhaps the case to insist a little bit longer on this issue, in order to clarify a few points to be understood in the way we intend to explain them.

In the general case, the integrand in Equation (A1) has the form [23]

(A3) $$\left\langle d\Phi |d\Phi \right\rangle \equiv g_{\alpha \beta }\frac{\partial \Phi^{\alpha }}{\partial x^{i}}\frac{\partial \Phi^{\beta }}{\partial x^{j}}h^{ij}$$

where the summation convention applies on all indices. Here, g is the metric in the space of mappings Φ, whose components are indexed by Greek indices, and h is the metric in the ambient space, for which we use the Latin indices. In the cases where h is Euclidean and g is the Poincaré metric, we have the expression of the integrand as written in Equation (A2).

The original theory of Skyrme does not take this “already built-in” aspect of the classical model of the atom into consideration. Rather, the nucleus is assumed to have a particulate structure in the regular Euclidean 4D space, and in this case the harmonic principle leads to a linear equation. Indeed, in this case we have to manage with the Euclidean 3-sphere, and the mapping Φ is given by the normal vector of the unit sphere, $\vec{x}$ say. Therefore, Equation (A3) takes the form

(A4) $$\left\langle d\Phi |d\Phi \right\rangle \equiv \delta_{\alpha \beta }\frac{\partial n^{\alpha }}{\partial x^{i}}\frac{\partial n^{\beta }}{\partial x^{j}}\delta^{ij}=\sum_{\alpha }\left(\nabla n^{\alpha }\cdot \nabla n^{\alpha }\right)$$

Thus, the differential equation corresponding to the functional (A1) is linear: the vector $\vec{n}$ has as components solutions of the Laplace equation. In view of this linearity of the resulting equation, if not somehow amended, the harmonic principle cannot account for the particulate structure, which here would occur in the classical form of topological solitons. Therefore, it appears that the Skyrme original functional, which was formulated in terms of deformations of a regular Euclidean space [24], is not quite as simple as the above one, but involves higher-degree terms belonging to a different cohomology class and given by the equation

(A5) $$E_{4}\left(\Phi \right)=∭\left\langle d\Phi \wedge d\Phi |d\Phi \wedge d\Phi \right\rangle d^{3}\vec{x}$$

where ∧ is the exterior product of the differentials. Incidentally, the indices 2 and 4 occurring in the energetic functionals of Skyrme are justified by the orders of the tensors entering the integrands of those functionals. In this case, we must first construct the 2-forms entering the interior product. They are defined according to the matrix multiplication of dΦ, so that in the previous situation, we will have

(A6) $$d\Phi \wedge d\Phi \equiv \nabla n^{\alpha }\times \nabla n^{\beta }$$

This time, however, one has to manage with a skew symmetric tensor in the upper indices, and the internal product from Equation (A5) is largely a matter of choice. The usual choice is the trace of the square of the tensor:

(A7) $$\left\langle d\Phi \wedge d\Phi |d\Phi \wedge d\Phi \right\rangle =\sum_{\alpha }{\left(\nabla n^{\alpha }\times \nabla n^{\beta }\right)}^{2}$$

Thus, the actual Skyrme functional corresponding to a situation describing the nuclear matter was originally written in an algebraically non-homogeneous form, something like

(A8) $$E\left(\Phi \right)=\frac{1}{2}∭\left(a\left\langle d\Phi |d\Phi \right\rangle +b\left\langle d\Phi \wedge d\Phi |d\Phi \wedge d\Phi \right\rangle \right)d^{3}\vec{x}$$

and is referring to a mapping Φ from the real space to itself. This is now determined as a solution of the nonlinear differential equation resulting from the variational principle which refers to the functional (A8). This equation is

(A9) $$a\nabla^{2}n^{\alpha }+n_{\beta }\left(n^{\alpha }\times n^{\beta }\right)=0$$

In a particular case, Skyrme presents a “static solution” in the form of the “hedgehog ansatz”, representing a nucleon:

(A10) $$\Phi \left(\vec{r}\right)=\exp\left\{\left(\vec{\tau }\cdot \vec{r}\right)F\left(r\right)\right\}$$

where $\vec{\tau }$ is the triple of Pauli matrices representing the nuclear isospin. The Equation (A9) then actually turns into a differential equation for the function $F\left(r\right)$, which can be solved in different assumptions. Almost any variation in the Skyrme model, including the original one of Skyrme, who introduced the baryonic number as a topological invariant, takes this basic situation for granted. The exceptions are a few approaches that reflect the indecision of the model outside the realm of the mappings from ordinary Euclidean space to itself.

There are, however, works that touch our own subject matter here, and quite explicitly at that. One of these seems, for the moment, worth mentioning again, for it guides our guesses. In their work from 2004, Atiyah and Sutcliffe [25] seem to point out the fundamental significance of hyperbolic skyrmions. A conclusion of this work is indeed especially noticeable, namely that the theory of Euclidean skyrmions with massive pions leads to detailed calculation results that are almost identical to those referring to *massless hyperbolic skyrmions*.

It is thus symptomatic that a structure of space per se, which is almost of no concern in the alike problem of general relativity, for instance, kicks back into a theory that seems so far away from it. In this respect, it is perhaps worth recalling that the vacuum or electrovacuum Einstein equations are equivalent with the harmonic principle represented by the metric from Equation (A2). The resulting differential equation is actually “already” nonlinear, leading directly to solitons (see [26], especially Section 8). It is this aspect of the theory of hyperbolic skyrmions which, when combined with the results of Atiyah and Sutcliffe, seems to lead to a promising theoretical understanding of the nuclear matter. From this perspective, it seems just a mishap that the theory of Euclidean skyrmions appears to be completely disjointed from that of the hyperbolic skyrmions. However, it is indeed just an appearance, inasmuch as in going along the line of Euclidean skyrmions one of the most notable results has emerged, which gives us the possibility to put the hyperbolic skyrmions on a sound physical basis, on a par we might say, and with the very help of the Euclidean skyrmions [27].

## Appendix B

The multifractal Schrödinger equation admits a specific SL(2,R) group [28,29]. Working in the variables $\left(t,r\right)$, the finite equations of this group are given by

(A11) $$t\to \frac{\alpha t+\beta }{\gamma t+\delta };\;r\to \frac{r}{\gamma t+\delta }$$

Then, in the tangent space SL(2,R), every vector is a linear combination of three fundamental vectors:

(A12) $$X_{1}=\frac{\partial }{\partial t},\;\;X_{2}=t\frac{\partial }{\partial t}+\frac{r}{2}\frac{\partial }{\partial r},\;\;X_{3}=t^{2}\frac{\partial }{\partial t}+tr\frac{\partial }{\partial r}$$

satisfying the basic structure equations:

(A13) $$\left[X_{1},X_{2}\right]=X_{1},\;\;\left[X_{2},X_{3}\right]=X_{3},\;\;\left[X_{3},X_{3}\right]=-2X_{2}$$

which we take as standard commutation relations for this type of algebraic structure throughout the present work.

Now, consider the firs part of Equation (A11), which represents the homographic action of the generic matrix that we denote by $\hat{M}$:

(A14) $$\hat{M}=\left(\begin{array}{cc} \alpha & \beta \\ \gamma & \delta \end{array}\right)$$

The problem that we want to solve is the following: to find the relationship between the set of matrices and a set of values of t for which t remains constant. From a geometrical point of view this means finding the set of points $\left(\alpha ,\beta ,\gamma ,\delta \right)$ that uniquely correspond to the values parameter t. Using the first part of Equation (A11), our problem is solved by a Riccati differential equation that is obtained as a consequence of the constancy of t:dt=0

(A15) $$dt+\omega_{1}t^{2}+\omega_{2}t+\omega_{3}=0$$

where we use the following notations:

(A16) $$\begin{array}{ll} \omega_{1} & =\frac{\gamma d\alpha -\alpha d\gamma }{\alpha \delta -\beta \gamma }\\ \omega_{2} & =\frac{\delta d\alpha -\alpha d\delta +\gamma d\beta -\beta d\gamma }{\alpha \delta -\beta \gamma }\\ \omega_{3} & =\frac{\delta d\beta -\beta d\delta }{\alpha \delta -\beta \gamma } \end{array}$$

The three differential forms in Equations (A16) constitute what is commonly known as a coframe at any point of absolute space. This coframe allows us to translate the geometric properties of absolute space into algebraic properties related to differential Equation (A15).

The simplest of these properties refer to the motion on geodesics of the metric

(A17) $$ds^{2}=\frac{1}{4}\left[{\left(\omega_{2}\right)}^{2}-4\omega_{1}\omega_{3}\right]$$

In this case, the 1-forms $\omega_{1}$, $\omega_{2}$, $\omega_{3}$ are exact differentials in the same parameter of the length of the arc of the geodesic, let us say. Along these geodesics, Equation (A15) turns into an ordinary differential equation of the Riccati type:

(A18) $$\frac{dt}{ds}=a_{1}t^{2}+2a_{2}t+a_{3}$$

Here, the parameters $a_{1,2,3}$ are constants that characterize a certain geodesic of the family.

For example, the absolute metric for the interior of the circle $\mu^{2}+\nu^{2}=1$

(A19) $$ds^{2}=\frac{\left(1-\nu^{2}\right){\left(d\mu \right)}^{2}+2\mu \nu \left(d\mu \right)\left(d\nu \right)+\left(1-\mu^{2}\right){\left(d\nu \right)}^{2}}{{\left(1-\mu^{2}-\nu^{2}\right)}^{2}}$$

can be brought to the form of Poincaré metric

(A20) $${\left(ds\right)}^{2}=-4\frac{dh\cdot dh^{*}}{{\left(h-h^{*}\right)}^{2}}=\frac{{\left(du\right)}^{2}+{\left(dv\right)}^{2}}{v^{2}}$$

by the following transformation of coordinates:

(A21) $$\mu =\frac{hh^{*}-1}{hh^{*}+1},\;\nu =\frac{h+h^{*}}{hh^{*}+1}↔h\equiv u+iv=\frac{v+i\sqrt{1-\mu^{2}-\nu^{2}}}{1-\mu };\;h^{*}=u-iv$$

The conservation laws for the metric (A20) are represented by the following differential 1-forms:

(A22) $$\omega_{1}=\frac{du}{v^{2}},\;\;\omega_{2}=1\frac{udu+vdv}{v^{2}},\;\;\omega_{3}=\frac{\left(u^{2}-v^{2}\right)du+2uvdv}{v^{2}}$$

In such context, if we consider a Levi-Civita parallel transport of direction [2,3]:

(A23) $$d\theta =-\frac{du}{v}$$

where Equation (A20) can be written as a sum of perfect squares:

(A24) $$ds^{2}=d\theta^{2}+{dln\left(v\right)}^{2}$$

From here, a Hamiltonian theory can be built.

## Appendix C

We can consider that any complex system can be assimilated to a mathematical multifractal-type object. Then, in the framework of the Scale Relativity Theory [1,2,3], the dynamics of any complex system can be described through the multifractal scale covariance derivative [3], where

(A25) $$\frac{\hat{d}}{dt}=\partial_{t}+{\hat{V}}^{l}\partial_{l}+\frac{1}{4}{\left(dt\right)}^{\left[\frac{2}{f\left(\alpha \right)}\right]-1}D^{lp}\partial_{l}\partial_{p}$$

(A26) $${\hat{V}}^{l}=V_{D}^{l}-iV_{F}^{l}$$

(A27) $$D^{lp}=d^{lp}-i{\hat{d}}^{lp}$$

(A28) $$d^{lp}=\lambda_{+}^{l}\lambda_{+}^{p}-\lambda_{-}^{l}\lambda_{-}^{p}$$

(A29) $${\hat{d}}^{lp}=\lambda_{+}^{l}\lambda_{+}^{p}+\lambda_{-}^{l}\lambda_{-}^{p}$$

(A30) $$\partial_{t}=\frac{\partial }{\partial t},\;\;\;\;\;\;\;\;\;\partial_{l}=\frac{\partial }{\partial x^{l}},\;\;\;\;\;\;\;\;\;\partial_{l}\partial_{p}=\frac{\partial }{\partial x^{l}}\frac{\partial }{\partial x^{p}},\;\;\;\;\;\;\;\;\;i=\sqrt{-1},\;\;\;\;\;\;\;\;\;l,\;\;\;\;\;\;\;\;\;p=1,2,3$$

The meaning of the quantities from (A25)–A(30) are given in [1,2,3].

Now, accepting the functionality of the multiscale covariance principle (for more applications in relevant domains, see [1,2,3]), which entails the application of the operator (A25) to the complex velocity fields (A26), without any external constraint, the motion equations of the polymer–drug structural unit dynamics take the following form:

(A31) $$\frac{d{\hat{V}}^{i}}{dt}=\partial_{t}{\hat{V}}^{i}+{\hat{V}}^{l}\partial_{l}{\hat{V}}^{i}+\frac{1}{4}{\left(dt\right)}^{\left[\frac{2}{f\left(\alpha \right)}\right]-1}D^{lk}\partial_{l}\partial_{k}{\hat{V}}^{i}=0$$

This means that the multifractal acceleration, $\partial_{t}{\hat{V}}^{i}$, the multifractal convection, ${\hat{V}}^{l}\partial_{l}{\hat{V}}^{i}$, and the multifractal dissipation, $D^{lk}\partial_{l}\partial_{k}{\hat{V}}^{i}$, achieve a balance at every point of any multifractal curve of the complex system structural units’ dynamics. From here, separating the complex system structural units’ dynamics by scale resolution (differentiable and non-differentiable scale resolutions), (A31) becomes

(A32) $$\partial_{t}V_{D}^{i}+V_{D}^{l}\partial_{l}V_{D}^{i}-V_{F}^{l}\partial_{l}V_{F}^{i}+\frac{1}{4}{\left(dt\right)}^{\left[\frac{2}{f\left(\alpha \right)}\right]-1}D^{lk}\partial_{l}\partial_{k}V_{D}^{i}=0\;\partial_{t}V_{F}^{i}+V_{F}^{l}\partial_{l}V_{D}^{i}+V_{D}^{l}\partial_{l}V_{F}^{i}-\frac{1}{4}{\left(dt\right)}^{\left[\frac{2}{f\left(\alpha \right)}\right]-1}D^{lk}\partial_{l}\partial_{k}V_{F}^{i}=0$$

According to [4], the main multifractalization route is through stochasticization. Thus, in the following, the case of multifractalization by Markov-type stochastic processes, which implies the conditions [1,2,3,4], will be considered:

(A33) $$\lambda_{+}^{i}\lambda_{+}^{l}=\lambda_{-}^{i}\lambda_{-}^{l}=2\lambda {\left(dt\right)}^{\left[\frac{2}{f\left(\alpha \right)}\right]-1}\delta^{il},\;\;\;\;\;i,l=1,\;2,\;3$$

where $\lambda {\left(dt\right)}^{\left[\frac{2}{f\left(\alpha \right)}\right]-1}$ are specific coefficients associated with the transition from differentiable to non-differentiable dynamics (which are actually differentiable–non-differentiable scale transitions) and $\delta^{il}$ is the Kronecker pseudo-tensor. Considering the functionality of (A29) in the drug-release dynamics, (A31) takes the form

(A34) $$\frac{\hat{d}{\hat{V}}^{i}}{dt}=\partial_{t}{\hat{V}}^{i}+{\hat{V}}^{l}\partial_{l}{\hat{V}}^{i}-i\lambda {\left(dt\right)}^{\left[\frac{2}{f\left(\alpha \right)}\right]-1}\partial_{l}\partial^{l}{\hat{V}}^{i}=0$$

and, from here, by separating the complex system dynamics on scale resolutions, we obtain

(A35) $$\partial_{t}V_{D}^{i}+V_{D}^{l}\partial_{l}V_{D}^{i}-\left[V_{F}^{l}+\lambda {\left(dt\right)}^{\left[\frac{2}{f\left(\alpha \right)}\right]-1}\partial^{l}\right]\partial_{l}V_{F}^{i}=0\;\partial_{t}V_{F}^{i}+V_{D}^{l}\partial_{l}V_{F}^{i}+\left[V_{F}^{l}+\lambda {\left(dt\right)}^{\left[\frac{2}{f\left(\alpha \right)}\right]-1}\partial^{l}\right]\partial_{l}V_{D}^{i}=0$$

For the irrotational motion of the complex system structural units’ dynamics, the complex velocity field (A26) takes the form

(A36) $${\hat{V}}^{i}=-2i\lambda (dt{)}^{\left[\frac{2}{f\left(\alpha \right)}\right]-1}\partial^{i}\ln\Psi$$

where Ψ is the state function.

Now, by substituting (A36) in (A34), based on the mathematical procedure from [1,2,3], the Schrödinger multifractal equations results:

(A37) $$\alpha^{2}\partial_{l}\partial^{l}\Psi +i\alpha \partial_{t}\Psi +\frac{U}{2}\Psi =0$$

where

(A38) $$\alpha =\lambda {\left(dt\right)}^{\left[\frac{2}{f\left(\alpha \right)}\right]-1}$$

## References

[1] Nottale L. (2011). Scale Relativity and Fractal Space-Time: A New Approach to Unifying Relativity and Quantum Mechanics.

[2] Merches I., Agop M. (2016). Differentiability and Fractality in Dynamics of Physical Systems.

[3] Agop M., Paun V.P. (2017). On the new perspectives of fractal theory. Fundaments and Applications.

[4] Mandelbrot B.B. (2004). Fractal and Chaos.

[5] Cristescu C.P. (2008). Nonlinear dynamics and chaos. Theoretical Fundaments and Applications.

[6] Farhi E., Khoze V.V., Singleton R. (1993). Minkowski space non-Abelian classical solutions with noninteger winding number change. Phys. Rev. D.

[7] Taraboanta I., Stoleriu S., Nica I., Georgescu A., Gamen A.C., Maftei G.A., Andrian S. (2020). Roughness variation of a nanohybrid composite resin submitted to acid and abrasive challenges. Int. J. Med. Dent..

[8] Iovan G., Stoleriu S., Nica I., Solomon S., Munteanu A., Andrian S. (2016). Surface characteristics of restorative composite resins after polishing with profine lamineer tips. Mater. Plast..

[9] Tofan N., Andrian S., Nica I., Stoleriu S., Topoliceanu C., Chelariu R., Bolat M., Pancu G. (2016). The Assessment of Erosive Potential of Some Acid Beverages on Indirect—Restorative Materials. Rev. Chim..

[10] Pancu G., Iovan G., Ghiorghe A., Topoliceanu C., Nica I., Tofan N., Stoleriu S., Sandu A.V., Andrian S. (2015). The assessment of biological parameters and remineralisation potential of saliva in pregnancy. Rev. Chim..

[11] Stoleriu S., Iovan G., Pancu G., Nica I., Andrian S. (2013). Study concerning the influence of the finishing and polishing systems on the surface state of various types of composite resins. Rom. J. Oral Rehabil..

[12] Mazilu N., Agop M., Gatu I., Iacob D.D., Ghizdovăt V. (2016). From Kepler problem to skyrmions. Mod. Phys. Lett. B.

[13] Dittrich W., Reuter M. (2017). Classical, and Quantum Dynamics.

[14] Zelikin M.I. (2000). Control Theory and Optimization, Encyclopaedia of Mathematical Sciences.

[15] Denman H.H. (1968). Time Translation Invariance for Certain Dissipative Classical Systems. Am. J. Phys..

[16] Landau L.D., Lifschitz E.M. (1966). Mécanique, Mir Editions, Moscou Larmor, J. (1900): Aether and Matter.

[17] Carinena J.F., Ramos A. (1999). Integrability of the Riccati equation from a group-theoretical viewpoint. Int. J. Mod. Phys. A.

[18] Stoler D. (1970). Equivalence Classes of Minimum Uncertainty Packets. Phys. Rev..

[19] Stoler D. (1971). Generalized Coherent States. Phys. Rev..

[20] Skyrme T.H.R. (1988). The Origins of Skyrmions. Int. J. Mod. Phys..

[21] Skyrme T.H.R., Brown G.E. (1994). Selected Papers, with Comentary.

[22] Slobodeanu R. (2009). On the Geometrized Skyrme and Faddeev Models. arXiv.

[23] Misner C.W. (1978). Harmonic Maps as Models for Physical Theories. Phys. Rev. D.

[24] Skyrme T.H.R. (1961). A Non-Linear Field Theory. Proc. R. Soc. Lond..

[25] Atiyah M.F., Sutcliffe P. (2004). Skyrmions, Instantons, Mass and Curvature. Phy. Lett. D.

[26] Rogers C., Schief W.K. (2002). Bäcklund and Darboux Transformations.

[27] Canfora F., Maeda H. (2013). Hedgehog ansatz and its generalization for self-gravitating Skyrmions. Phys. Rev. D.

[28] Niederer U. (1972). The Maximal Kinematical Invariance Group of the Free Schrödinger Equation. Helv. Phys. Acta.

[29] De Alfaro V., Fubini S., Furlan G. (1976). Conformal Invariance in Quantum Mechanics. Nuovo Cimento A.

